# Positive Effects of the Nutraceutical Association of Lycopene and Selenium in Experimental Varicocele

**DOI:** 10.3390/ijms241713526

**Published:** 2023-08-31

**Authors:** Jose Freni, Giovanni Pallio, Herbert Ryan Marini, Antonio Micali, Natasha Irrera, Carmelo Romeo, Domenico Puzzolo, Federica Mannino, Letteria Minutoli, Igor Pirrotta, Alessandro Scarfone, Pietro Antonuccio

**Affiliations:** 1 Department of Biomedical, Dental Sciences and Morphofunctional Imaging, University of Messina, 98125 Messina, Italy; jose.freni@unime.it (J.F.); puzzolo@unime.it (D.P.); 2 Department of Clinical and Experimental Medicine, University of Messina, 98125 Messina, Italy; giovanni.pallio@unime.it (G.P.); hrmarini@unime.it (H.R.M.); nirrera@unime.it (N.I.); federica.mannino@unime.it (F.M.); ipirrotta@unime.it (I.P.); alessandro.scarfone@unime.it (A.S.); 3 Department of Human Adult and Childhood Pathology, University of Messina, 98122 Messina, Italy; amicali@unime.it (A.M.); romeoc@unime.it (C.R.); pietro.antonuccio@unime.it (P.A.)

**Keywords:** testis, varicocele, selenium, lycopene, diet, nutraceuticals, testosterone, caspase-3, TUNEL assay, HIF-1α

## Abstract

Many natural substances commonly found in healthy diets have been studied for their potential to reduce male infertility associated with varicocele. A positive role of selenium (Se) or lycopene alone was demonstrated in experimental varicocele, while no data are available on their association. One group of male Sprague–Dawley rats was sham operated and daily treated with Se (3 mg/kg, i.p.), lycopene (1 mg/kg, i.p.), or their association. A second group underwent surgery to induce varicocele. Sham and half of the varicocele animals were sacrificed after twenty-eight days, while the residual animals were treated for one more month and then sacrificed. In varicocele animals, testosterone levels and testes weight were reduced, Hypoxia Inducible Factor-1α (HIF-1α) expression was absent in the tubules and increased in Leydig cells, caspare-3 was increased, seminiferous epithelium showed evident structural changes, and many apoptotic germ cells were demonstrated with TUNEL assay. The treatment with lycopene or Se alone significantly increased testis weight and testosterone levels, reduced apoptosis and caspase-3 expression, improved the tubular organization, decreased HIF-1α positivity of Leydig cells, and restored its tubular positivity. Lycopene or Se association showed a better influence on all biochemical and morphological parameters. Therefore, the nutraceutical association of lycopene plus Se might be considered a possible therapeutic tool, together with surgery, in the treatment of male infertility. However, long-term experimental and clinical studies are necessary to evaluate sperm quantity and quality.

## 1. Introduction

A varicocele is characterized by the elongation and widening of the internal testicular vein and the spermatic pampiniform plexus, often leading to male infertility. It is caused either by an abnormal position of the left spermatic vein, by a dysfunction of venous valves, or by anomalies of the left renal vein, all inducing a hindered spermatic venous reflux [1].

An analysis of the literature showed an overall prevalence of 15–20% in the healthy adult male population. In patients with male infertility, varicocele was demonstrated in 20–40% of subjects with primary infertility and in 75–81% of men with secondary infertility. Typically, left-sided varicoceles are present in 90% of cases and only 10% are bilateral [1].

The hypotheses of varicocele contribution to infertility include higher temperature in scrotum, hypoxia, oxidative stress, accumulation of carbon dioxide, apoptosis, inflammation, autoimmunity, and damage to sperm cells, but no consensus theory is able to explain the effects of varicocele on infertility [2].

While the full impact of varicocele on infertility remains unclear, oxidative stress and testicular apoptosis are believed to play significant roles. Under hypoxic conditions, an increased production of reactive oxygen species (ROS) occurs, inducing negative changes of the plasma membranes of spermatozoa and of the other cells of the germinal epithelium and stimulating testicular apoptosis [3,4]. This latter process can also be correlated to the increased expression of Hypoxia Inducible Factor-1α (HIF-1α) [5,6].

HIF-1, a nuclear transcription factor, is present either in normoxic conditions or under hypoxia [6]. In particular, HIF-1α, the active subunit of HIF-1, regulates the physiological activity and the expression of this factor. In testis, HIF-1α is present under both normoxia and ischemia [7]. An evident increase in its expression was shown in experimental models of ischemia/reperfusion [7,8], even if its role has not yet been completely elucidated.

The outcomes of different substances on the biochemical and morphological damages induced in testes by experimental varicocele were studied in the last years [7,9,10,11,12,13,14,15]. In particular, the effects of lycopene and selenium (Se) were evaluated. Indeed, in recent years, human clinical research indicated that nutritional interventions, based on lycopene and Se, substances well represented in healthy dietary habits, can significantly counteract male infertility [9,10].

Lycopene is considered the strongest anti-radical compound of the carotenoid family [16] and can be found in vegetables and fruits, such as tomatoes, papayas, watermelons, apricots, and pink grapefruits [17]. Lycopene is accumulated in the testes, where its levels are ten-fold greater than in other organs, owing to a large number of receptors for lipoproteins or a higher uptake of lipoproteins [12]. In experimental varicocele, lycopene showed antioxidant and antiapoptotic activity, as it significantly improved testes weight, testosterone levels, B-cell lymphoma 2 (Bcl-2) expression, seminiferous tubules organization and decreased ROS levels, BAX expression, and the number of terminal deoxynucleotidyl transferase dUTP nick end labeling (TUNEL)-positive cells [10,12].

Se, an essential trace element, plays significant roles in many biochemical and physiological processes, including the reproductive function in males [9]. Its deficiency may cause morphological changes in the seminiferous tubules, with reduction in the number of spermatozoa and even reduced sperm motility [18]. In experimental varicocele, Se administration reduced oxidative stress, apoptosis, and morphological damages of seminiferous tubules [9,11]; however, biochemical and structural parameters were significantly different if compared to sham rats [11].

Interestingly, the role of the association between lycopene and Se was not evaluated in experimental varicocele, even if our group has previously examined its positive effects in benign prostatic hypertrophy [19]. As to the mechanism of action of lycopene and Se, especially in the context of reproductive health, specific morphological and biochemical parameters are provided, even if further long-term evaluations on the quantity and quality of sperm are necessary.

On the basis of this background, the aim of the present work is to better elucidate the role of the nutraceutical association of lycopene and Se in an experimental model of varicocele in rats.

## 2. Results

### 2.1. Influence of Lycopene and Se Alone and Their Association on Testis Weight

Table 1 shows the results of testis weight in all the examined groups. All sham groups had superimposable weight, so that a single value is indicated as representative of the sham. In varicocele rats, the weight of operated and contralateral testes was significantly lower than the sham groups, but contralateral testes had a greater weight if compared to operated. In both varicocele plus lycopene alone and varicocele plus Se alone treated rats, the weight of operated and contralateral testes was significantly lower than sham rats, even if contralateral testes exhibited higher values. In varicocele rats treated with lycopene plus Se, both operated and contralateral testes showed a close to sham weight.

### 2.2. Influence of Lycopene and Se Alone and Their Association on Testosterone Levels

All sham groups showed normal levels of testosterone; consequently, a single value is provided for sham. A sharp, significant decrease was observed in varicocele animals when compared to sham (−54%). Administration of lycopene or Se alone caused an increase in testosterone levels compared to varicocele rats (+43% and +46%, respectively); however, the values were significantly lower than those of the sham groups. Only in varicocele rats treated with the association of lycopene plus Se were the testosterone levels close to sham (+54% compared to varicocele alone rats) (Table 1).

### 2.3. Influence of Lycopene and Se Alone and Their Association on Malondialdehyde (MDA) Levels

MDA levels were normal in all sham groups; therefore, we provided a single value for the sake of simplification. Conversely, MDA levels were significantly augmented in varicocele rats. When treated with lycopene or Se alone, MDA levels were significantly decreased in both varicocele and contralateral testes, but they were significantly higher than sham. Only in varicocele rats treated with lycopene and Se was MDA reduced to close to normal levels in either operated or contralateral testes (Table 1).

### 2.4. Influence of Lycopene and Se Alone and Their Association on Caspase-3 Expression

**Figure 1:**
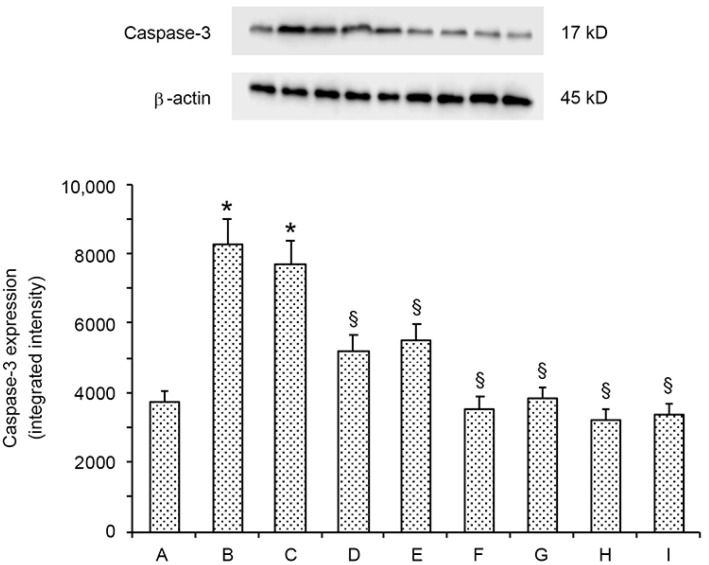
Representative Western blot analysis of caspase-3 in testes of sham (**A**); varicocele (**B**); varicocele contralateral (**C**); varicocele plus lycopene (**D**); varicocele plus lycopene contralateral (**E**); varicocele plus Se (**F**); varicocele plus Se contralateral (**G**); varicocele plus lycopene plus Se (**H**); and varicocele plus lycopene plus Se contralateral rats (**I**). * *p* ≤ 0.05 versus sham; § *p* ≤ 0.05 versus varicocele. Bars represent the mean ± SD of seven experiments.

A low expression of caspase-3 was present in all sham groups: therefore, a single value for sham is provided (Figure 1). Conversely, operated and contralateral testes of varicocele rats showed a significant increase (+2.1-fold and +1.9-fold, respectively) of caspase-3 expression. In the varicocele plus lycopene group, caspase-3 expression was significantly reduced in both operated and contralateral testes versus varicocele group (−1.5-fold and −1.2-fold, respectively). A further moderate decrease in caspase-3 expression was present in both operated and contralateral testes of varicocele plus Se rats (−2.2-fold and −1.8-fold, respectively). In varicocele plus lycopene and Se rats, caspase-3 expression was even lower, even if not significantly, than sham.

### 2.5. Administration of Lycopene and Se Alone and Their Association Counteracts Testes Changes

**Figure 2:**
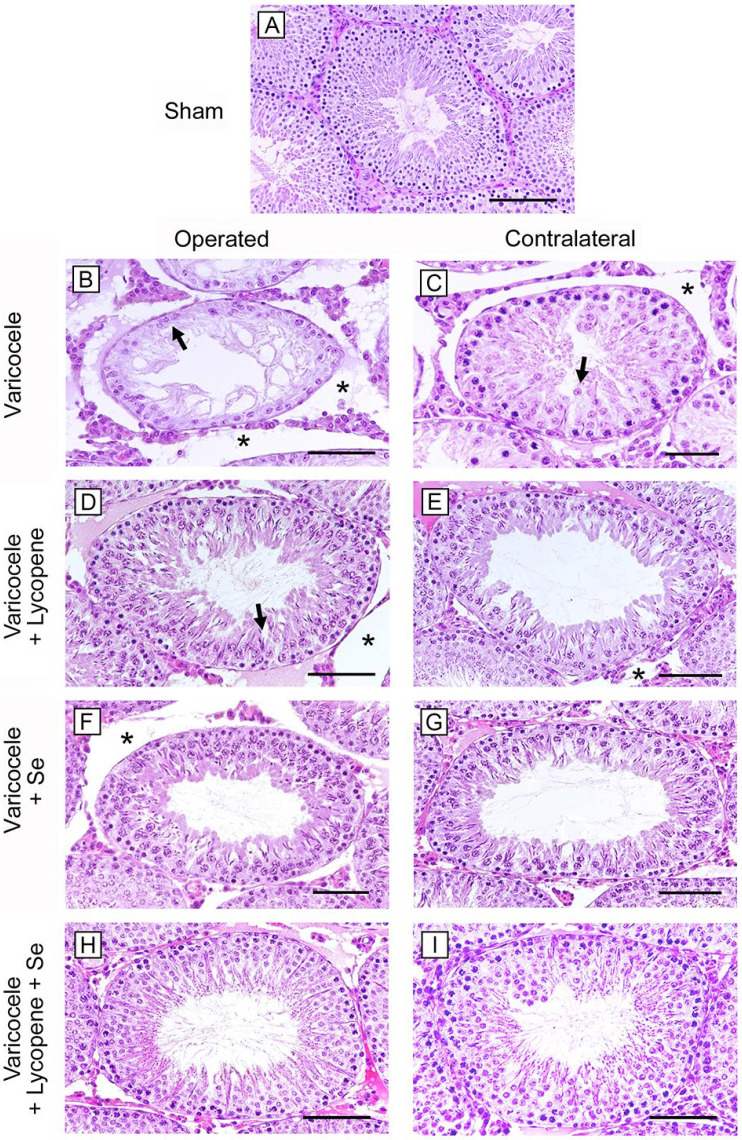
Histological organization of the testes (Hematoxylin–Eosin stain). (**A**): In rats of all the sham groups, a normal structure of both tubular and extratubular compartments is present. (**B**): In varicocele testes, a thin seminiferous epithelium with disorganized germ cells (arrow) and a marked edema (*) in the extratubular compartment can be observed. (**C**): In contralateral testes of the varicocele group, some spermatids and immature spermatozoa (arrow) and an evident edema (*) in the extratubular compartment are present. (**D**): In varicocele rats treated with lycopene, the seminiferous tubules are better preserved, even if intercellular clefts are present (arrow). The extratubular compartment is edematous (*). (**E**): In contralateral testes of the same group, the structural organization of the seminiferous tubules is improved, and the extratubular compartment shows a mild edema (*). (**F**): In varicocele rats treated with Se, the tubules show a close to normal organization, but an interstitial edema (*) is present. (**G**): In the contralateral testes of the same group, both the tubules and the extratubular compartment are close to normal. (**H**,**I**): In varicocele rats treated with lycopene and Se, in both operated and contralateral testes, the tubular and the interstitial structure is close to sham. (Scale bar: (**A**,**D**–**I**) = 100 µm; (**B**,**C**) = 50 µm).

In animals of all the sham groups, a normal morphology of the seminiferous tubules and of the extratubular compartment was observed with Hematoxylin and Eosin (HE) stain. Therefore, a single image is provided as representative of sham (Figure 2A) and only one datum is provided for mean tubular diameter (MTD) and Johnsen’s score (JS) (Table 2). In the seminiferous tubules of varicocele rats, an atrophic epithelium with disorganized germ cells was demonstrated; in the adluminal compartment, condensed sperm tails were observed (Figure 2B). Consequently, the tubules showed a sharp reduction of their MTD and low JS (Table 2). A marked edema was present in the extratubular compartment. In contralateral testes of the same varicocele group (Figure 2C; Table 2), the mean tubular diameter was significantly reduced and some spermatids were present. In the extratubular compartment, an evident edema was detected. In varicocele rats treated with lycopene (Figure 2D; Table 2), the seminiferous tubules were better preserved, even if intercellular clefts were evident; MTD and JS were significantly lower when compared to sham rats. The extratubular compartment was edematous. In contralateral testes of the same group (Figure 2E; Table 2), the structural organization of the seminiferous tubules was close to normal, as indicated also by MTD and JS, and the extratubular compartment showed a mild edema. In varicocele rats treated with Se (Figure 2F; Table 2), MTD and JS were still significantly lower than sham, and the extratubular compartment showed an interstitial edema. In the contralateral testes of the same group (Figure 2G; Table 2), the structural organization of the tubules and the extratubular compartment were close to normal, as indicated by the significantly higher MTD and JS; in varicocele rats treated with lycopene and Se, in both operated and contralateral testes (Figure 2H,I; Table 2), a structural organization, an MTD, and a JS close to sham were observed.

### 2.6. Influence of Lycopene and Se Alone and Their Association on Apoptosis with TUNEL Assay

**Figure 3:**
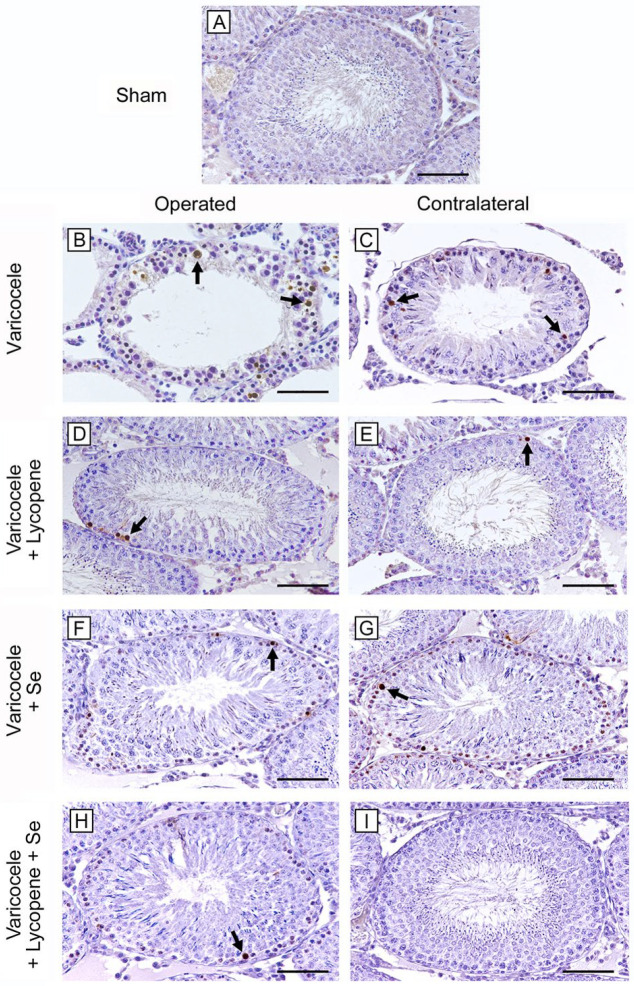
Assessment of apoptosis with TUNEL stain in the different groups of rat testes. (**A**): In the testes of all sham rats, no TUNEL-positive cells are present. (**B**,**C**): In both varicocele and CL testes, many TUNEL-positive cells (arrow) are evident, being more numerous in operated rats. (**D**,**E**): In varicocele and in CL testes of rats treated with lycopene alone, TUNEL-positive cells (arrow) are significantly reduced compared with varicocele rats. (**F**,**G**): In varicocele and in CL testes of rats treated with Se alone, few TUNEL-positive peripheral germ cells (arrow) are present. (**H**,**I**): In varicocele rats treated with lycopene and Se, both operated and CL testes showed only occasional TUNEL-positive spermatogonia (arrow). (Scale bar: (**A**,**D**–**I**) = 100 µm; (**B**,**C**) = 50 µm).

No TUNEL-positive cells were demonstrated in the seminiferous tubules of all groups of sham rats; therefore, only one micrograph is shown as typical of all groups (Figure 3A). Conversely, in varicocele testes, seminiferous tubules showed a large number of TUNEL-positive cells (Figure 3B). In fact, TWAC and the apoptotic index were significantly higher if compared to sham group (Table 2). In varicocele CL testes, many TUNEL-positive spermatogonia were present (Figure 3C), even if TWAC and the apoptotic index were significantly lower than operated testes (Table 2). In varicocele and in CL testes of rats treated with lycopene alone, the number of TUNEL-positive cells was significantly reduced, if compared with varicocele rats (Figure 3D,E). TWAC and the apoptotic index were significantly decreased, but higher than sham (Table 2). In varicocele and in CL testes of rats treated with Se alone, few TUNEL-positive peripheral germ cells were observed (Figure 3F,G), but both TWAC and the apoptotic index values were significantly higher than sham (Table 2). In varicocele rats treated with lycopene and Se, only occasional TUNEL-positive spermatogonia were present in both operated and CL testes (Figure 3H,I), even if TWAC and the apoptotic index in operated rats were significantly higher than sham (Table 2).

### 2.7. Influence of Lycopene and Se Alone and Their Association on HIF-1α Activity

**Figure 4:**
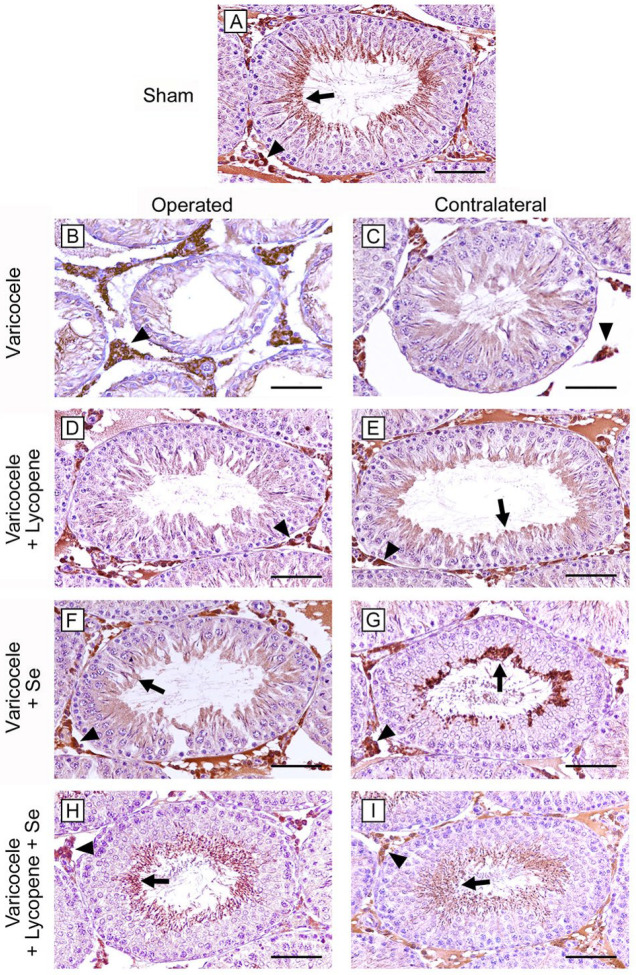
HIF-1α immunohistochemical expression in the different groups of rat testes. (**A**): In all sham groups, elongated spermatids and spermatozoa (arrow) and interstitial Leydig cells (arrowhead) are HIF-1α positive. (**B**,**C**): In varicocele rats, the highly damaged tubular wall of both operated and CL testes is HIF-1α negative; on the contrary, Leydig cells (arrowhead) are highly positive. (**D**): In the operated testes of varicocele plus lycopene group, the seminiferous epithelium is negative for HIF-1α, while Leydig cells (arrowhead) are positive. (**E**): In CL testes of varicocele plus lycopene treated rats, a mild HIF-1α positivity is present in spermatids (arrow), in addition to Leydig cells (arrowhead). (**F**): In operated testes of varicocele plus Se-treated group, a mild HIF-1α positivity is present in spermatids (arrow); Leydig cells (arrowhead) are positive. (**G**): In CL testes of varicocele plus Se treated rats, some spermatocytes and spermatids are HIF-1α positive (arrow), in addition to Leydig cells (arrowhead). (**H**,**I**): In the seminiferous tubules of both operated and CL testes of varicocele plus lycopene plus Se-treated rats, elongated spermatids and spermatozoa (arrow) and Leydig cells (arrowhead) are HIF-1α positive. (Scale bar: (**A**,**D**–**I**) = 100 µm; (**B**,**C**) = 50 µm).

All sham groups showed seminiferous tubules with HIF-1α positive elongated spermatids and spermatozoa; HIF-1α positive Leydig cells were also present in the interstitial spaces. Consequently, a single micrograph is shown as typical of all shams (Figure 4A). On the contrary, no positivity for HIF-1α was observed in the greatly damaged seminiferous tubules of both operated and CL testes of varicocele rats, while a higher expression was present in Leydig cells when compared to sham rats (Figure 4B,C). No positivity for HIF-1α was observed in the seminiferous epithelium of the operated rats treated with lycopene, while positive interstitial cells were still present (Figure 4D). In CL testes of the same group (Figure 4E), a mild positivity of the luminal part of the seminiferous epithelium was observed. When operated testes of varicocele plus Se treated rats were considered, the seminiferous tubules showed HIF-1α positive cells, and the expression of Leydig cells was lower if matched to varicocele rats (Figure 4F). In CL testes of the same group, some spermatocytes and spermatids were HIF-1α positive (Figure 4G). In both operated and CL testes of varicocele rats challenged with lycopene and Se, HIF-1α positivity of elongated spermatids, spermatozoa, and Leydig cells was comparable to sham (Figure 4H,I).

## 3. Discussion

Varicocele is considered to be a cause of male infertility, able to induce abnormal semen with decreased motility of spermatozoa, low sperm count, and abnormal structure of the seminiferous epithelium [10,11,20]. Recent evidence suggested the pathophysiologic mechanisms of the disease, demonstrating that many factors, such as increased scrotal temperature, hypoxia, oxidative stress, augmented inflammatory cytokines, apoptosis, and hormonal imbalance are involved. However, the exact mechanism of the resulting infertility is not yet fully elucidated. As to its incidence, varicocele accounts for 20–40% in males with primary infertility, while, in subjects with secondary infertility, its occurrence is up to 80% [6].

Among the above mechanisms, testicular hypoxia plays a leading role in varicocele pathophysiology. In particular, under hypoxic conditions, HIF-1α, the active subunit of HIF-1, a highly specific nuclear transcription factor, is expressed, showing a relationship with the degree of hypoxia [6,21]. Previous studies demonstrated that a high expression of HIF-1α triggers the apoptosis pathway in asthenozoospermic patients with varicocele [5]. Similar data were obtained in experimental models of varicocele in rats, showing a tight correlation between this molecule and programmed cell death [22,23].

In a previous paper based on an experimental model of testicular ischemia/reperfusion in rats [7], we showed a significant increase in HIF-1α in Leydig cells. Similar results were obtained in the present paper, as we demonstrated. In varicocele testes, there was an increased expression of HIF-1α in Leydig cells, which could be related to the hypoxic conditions of the interstitial tissues in both experimental procedures. As a consequence, the increased expression of HIF-1α activates apoptosis in the seminiferous epithelium, as demonstrated by the increased expression of caspase-3 and the presence of TUNEL-positive cells. This can be attributed to a different behavior of Leydig and germinal cells. The former does not undergo apoptosis, probably owing to the possible activation, in hypoxic conditions, of antiapoptotic genes [7], while the increase in HIF-1α positivity in varicocele could be related to their close relationship to the experimentally hypoxic interstitial blood vessels. Germinal cells are located in two different environments, the basal and the luminal part of the tubules, separated by the blood–testis barrier formed by Sertoli cells [24]. In the basal compartment, O_2_ tension is higher than that of the luminal region, so that, under hypoxic conditions, HIF-1α produced by Leydig cells. It triggers apoptosis of spermatogonia, undergoing a self-renewal process [25]. On the contrary, in the luminal compartment, O_2_ tension is normally lower, as indicated by the basal expression of HIF-1α in response to the reduced access of O_2_ [26]. As a consequence, haploid male germ cells placed in the luminal part of the tubules are particularly sensitive to hypoxia induced by the experimental varicocele, undergoing dramatic structural changes.

As to the reduction in testosterone levels observed in the varicocele group, this could be related to the increase in HIF-1α in Leydig cells, indicating a hypoxic suffering of these cells, which is able to interfere with steroid synthesis in the interstitial cells. This is probably related to the reduced production of the steroidogenic acute regulatory (StAR) protein, which has an important role in the first step of testosterone and other steroid hormones synthesis [27].

Changes in testis weight are important parameters to analyze the effects of experimental procedures in laboratory animals. In our study, the varicocele group of rats showed reduced testicular weight when compared to the sham group, probably related to structural damage and to apoptosis of the germinal epithelium [27].

Another mechanism involved in varicocele is oxidative stress, mainly caused by an increased production of ROS [28], which interfere with lipids, proteins, and nucleic acids, inducing changes in the organization of seminiferous epithelium [10,11,29]. In our study, we observed an upregulation of the levels of MDA, the end product of lipid peroxidation, in the varicocele group. As a consequence, very evident structural changes were demonstrated in the tubules, consisting of an atrophic epithelium with disorganized germ cells and condensed sperm tails with a sharp reduction of MTD and JS. Furthermore, we found a marked edema in the extratubular compartment.

The main treatment of varicocele is varicocelectomy, which, particularly in adolescents, can ameliorate testicular growth and sperm characteristics. However, in the last two decades, in addition to surgery, the use of different substances, alone or in association, have been proposed in the management of the varicocele either in infertile men or in experimental animal models.

Among these substances, vitamins (C, D, E) [30,31,32,33], coenzyme Q10 [34], L-carnitine [35], zinc [36], magnesium [37], silymarin [38], berberine [39], thymoquinone [40], and resveratrol [41] were evaluated, showing a positive role for their antioxidant or antiapoptotic characteristics.

In addition, the effects of two nutraceuticals, lycopene and Se, alone or in association with different substances, were studied.

As to the lycopene, it is present in fruits and vegetables and is considered the most effective anti-free-radical of the carotenoid family [16]. Its distribution in the body is variable, being particularly concentrated in testes, adrenals, liver, and prostate [42]. From the evaluation of observational studies, a healthy diet rich in, among the other nutrients, lycopene was associated with high semen quality parameters [43]. Furthermore, in experimental varicocele, the administration of lycopene alone improved the quality of sperm owing to its antioxidant activity [10,12] and to the reduction of apoptosis [10,13,44]. No data are currently available on the association of lycopene with other substances in experimental varicocele.

As to Se, this essential and widely distributed element plays fundamental roles in the testis, as it is necessary for its normal development, for spermatogenesis, and for the normal activities of spermatozoa [45]. When administered to varicocele patients in association with other supplements [45,46,47,48], some significative improvements in sperm parameters were observed, which were related to the anti-oxidant activity of all the supplements [46]. A positive role of Se alone in reducing the oxidative status [9] and the number of apoptotic cells in the seminiferous tubules [11] was also observed in experimental varicocele.

Our paper shows, for the first time, the effects of the association of lycopene and Se in an experimental model of murine varicocele. We demonstrated an improvement in all biochemical and morphological parameters after the administration of both substances, thus indicating a positive role in fertility. This could be related to the involvement of HIF-1α, suggesting an important role of this transcriptional factor in hypoxic conditions of the testis. Indeed, as reported in a previous review by our research group, since the damage to testicular function is linked to multiple physio-pathological events, including testicular hypoxia and hyperthermia, inflammation, oxidative stress, hormonal imbalance, and cellular death, all substances tested in experimental varicocele, acting on this molecular context, could show synergistic effects, thus representing a promising and safe approach in this multifaceted pathology [49]. Specifically, these experimental data suggest a stronger anti-oxidant and anti-apoptotic action, directly or indirectly, on the above-mentioned molecular pathways by the lycopene and Se combination. Furthermore, the combination lycopene–Se showed neither side effects nor toxicity in our experimental protocol, even if long term studies or different associations are necessary for a better comprehension of possible interactions between these substances. As a matter of fact, other associations with Se or lycopene [7,19] in different experimental models were studied and showed no toxic effects.

Moreover, it appears consequential that healthy dietary habits, as plant-based diets (including the Mediterranean-style diet), which are particularly abundant in functional foods and/or nutraceuticals such as lycopene and Se, positively impact the above molecular targets deeply demodulated in varicocele [49].

## 4. Conclusions

The examined nutraceutical association of lycopene and Se could be included in the natural and safe management of varicocele and male infertility-related disorders, in support to surgery, even if additional studies are needed to evaluate further molecular targets involved in experimental varicocele. Long-term experimental and clinical studies are necessary to evaluate sperm quantity and quality after treatment with lycopene and Se. However, the pathophysiological knowledge of varicocele appears fascinating, but also deeply complex. As previously stated [49], and on the basis of the present experimental data, the development of an appropriate meta-analysis could be suitable to improve, from a translational point of view, our knowledge about the fascinating crosstalk between fertility and foods in varicocele patients.

## 5. Materials and Methods

### 5.1. Ethical Approval

In the present work we followed the standards for the care and use of animals, as indicated in the ARRIVE (Animal Research: Reporting In Vivo Experiments) guidelines [50]. Both the Italian Ministry of Health (authorization number 90/2017—PR) and the Institutional Animal Care and Use Committee of the University Hospital of Messina, Messina, Italy, approved all procedures.

### 5.2. Animals and Experimental Procedures

A total of 56 male Sprague–Dawley rats, aged 7 weeks and weighing 200–230 g, were purchased from Charles River Laboratories Italia srl (Calco, Italy). Animals were housed in controlled environmental conditions, under a cycle of 12-hour light/dark at approximately 23 °C, with food and water ad libitum. The animals were divided into 8 groups of 7 animals each. Four groups (n = 28; sham rats = control groups) were anesthetized and underwent a sham operation, as previously described [51]. The animals of the other four groups (n = 28; varicocele rats) were anesthetized, and varicocele was induced, as previously described in detail [52]. After 28 days from the surgical procedure, one group (sham or control) and one group (varicocele) were sacrificed. For the application of the principles of replacement, reduction, and refinement (the 3Rs) in animal research [53], and owing to the results of our previous experiments, we did not include in the present paper the groups with vehicles (corn oil and NaCl) of both sham and varicocele, as no changes were observed in testis morphology and in all the considered parameters [10,11]. The residual six groups (three sham or control groups and three varicocele groups) were treated daily for 30 days as follows: lycopene alone (1 mg/kg, i.p.), Se alone (3 mg/kg, i.p.), and lycopene (1 mg/kg, i.p.) plus Se (3 mg/kg, i.p.). The doses of both lycopene and Se were selected on the basis of the results of previous works [10,19]. After thirty days of treatment, all animals were euthanized with ketamine and xylazine (75/10 mg/kg, i.p., respectively). Blood was collected and both operated and contralateral testes were weighted and processed for biochemical, histopathological, and immunohistochemical evaluation.

### 5.3. Determination of Testosterone Levels

An ELISA kit was used for testosterone levels in serum, according to the protocol suggested by the manufacturer. In brief, blood, gained from cardiac puncture, was clotted, and serum was achieved by centrifugation for 10 min at 1000× *g*. An HRP-conjugate and the specific antibody were added, followed by substrates and stop solution. The mean absorbance was calculated using a microplate reader at 450 nm and correlated with those from standard curves. Data were expressed in ng/mL.

### 5.4. Determination of MDA Levels

Lipid peroxidation was evaluated through the MDA levels, indicating the results as nmol/g tissue [54].

### 5.5. Western Blot Analysis

Western blots were performed as previously described [7]. Specific primary antibody caspase 3 (Cleaved Caspase-3 Cod. #9664, Cell Signaling, Danvers, MA, USA) was diluted 1:1000 in 1X PBS, 5% *w*/*v* nonfat dried milk, 0.1% Tween-20, and incubated overnight at 4 °C. Then, the membrane was incubated with a secondary anti-rabbit (1:5000, GeneTex, Alton Pkwy Irvine, CA, USA) for 1 h at room temperature. To establish the loading of blots with equal quantities of protein lysates, it was also incubated with the antibody against β-actin (1:1000; GeneTex, Alton Pkwy Irvine, Irvine, CA, USA). Protein signal was analyzed by the enhanced chemiluminescence system (LumiGlo reserve; Seracare, Milford, MA, USA) and quantified by a scanning densitometry system (C-DiGit, Li-cor, Lincoln, NE, USA). Data were expressed as relative integrated intensity using β-actin (Cell Signaling, Danvers, MA, USA) as a control for the equal loading of samples.

### 5.6. Histological Evaluation

All testes, after fixation in Bouin, were dehydrated in ethanol, cleared in xylene, and embedded in paraffin (Paraplast, SPI Supplies, West Chester, PA, USA). Histological sections (5 µm) were mounted on silanized slides (Thermo Fisher Scientific, Waltham, MA, USA), and stained with HE. Photomicrographs were taken with a Nikon Ci-L (Nikon Instruments, Tokyo, Japan) light microscope, using a digital camera Nikon DS-Ri2, saved as Tagged Image Format Files (TIFF), and blindly assessed by two trained histologists. Five microscopic fields from ten non-serial sections of all groups, each including two entire seminiferous tubules, were evaluated. The diameters of 100 separate seminiferous tubules, all showing a circular profile, were calculated to obtain the MTD, expressed in micrometers (µm). From the same tubules, the structural organization of the germinal epithelium was assessed, according to the Johnsen’s scoring system [55], as modified by Erdemir et al. [56]. In brief, a score from 10 to 1 was assigned to each tubule on the basis of the organization of the seminiferous epithelium: 10: whole spermatogenesis and normal tubules; 9: many spermatozoa and disorganized spermatogenesis; 8: only a few spermatozoa; 7: no spermatozoa but many spermatids; 6: only a few spermatids; 5: no spermatozoa or spermatids but many spermatocytes; 4: only a few spermatocytes; 3: only spermatogonia; 2: no germ cells but only Sertoli cells; 1: no germ cells and no Sertoli cells.

### 5.7. TUNEL Assay for Apoptosis

A kit for the assay of apoptosis (In situ Apoptosis Detection kit, Abcam, Cambridge, UK) was used; 5 µm sections, obtained from the same samples used for morphological evaluation, were cleared in xylene and rehydrated in ethanol. Permeabilization with proteinase K was performed, followed by endogenous peroxidase blocking with 3% H_2_O_2_ in methanol. Sections were treated with terminal deoxynucleotidyl transferase, biotin-labeled deoxynucleotides, streptavidin–horseradish peroxidase conjugate, and then with diaminobenzidine as chromogen. Micrographs were taken with a Nikon Ci-L light microscope using a digital camera Nikon Ds-Ri2. One hundred seminiferous tubules per group were blindly evaluated by two trained observers to determine the percentage of tubules with apoptotic cells (%TWAC) and the mean number of TUNEL-positive cells per tubule (apoptotic index) [57].

### 5.8. Immunohistochemistry for HIF-1α

From the same blocks used for histological evaluation, 5 µm sections were mounted on silanized slides, cleared in xylene, and rehydrated in ethanol. Antigen retrieval was performed with pH 6.0 citrate buffer and endogenous peroxidase blocked with 0.3% H_2_O_2_ in methanol. Primary antibody (HIF-1α, 1:100, Orizor Scientific, Messina, Italy) was incubated overnight at 4 °C in a moisturized chamber the day after the secondary antibody (Pierce anti-rabbit, Cambridge, UK) was added. The reaction was visualized with 3,3′-Diaminobenzidine. Counterstaining was performed in Mayer’s haematoxylin. Slides were photographed with a Nikon Ci-L light microscope.

### 5.9. Statistical Analysis

The different experimental groups were analyzed by Student’s *t*-test and one-way ANOVA with Tukey’s post-test for intergroup comparisons. Values are expressed as mean ± standard deviation (SD). A *p* value of ≤ 0.05 was considered statistically significant.

## Figures and Tables

**Table 1:** Effects of Se (3 mg/kg/day), lycopene (1 mg/Kg/day), and Se (3 mg/kg/day) plus lycopene (1 mg/Kg/day) on testis weight, testosterone, and malondialdehyde (MDA) levels in varicocele rats as compared to sham and varicocele alone rats. Se = selenium; CL = contralateral testis. All values are expressed as mean ± SD; n = 7 animals for each group. ^a^ *p* < 0.05 vs. sham; ^b^ *p* < 0.05 vs. varicocele.

Groups	Testis Weight (g)	Testosterone (ng/mL)	MDA (nmol/g)
Sham	1.59 ± 0.12	5.9 ± 0.5	5.6 ± 0.5
Varicocele	0.81 ± 0.13 ^a^	2.7 ± 0.6 ^a^	12.1 ± 0.6 ^a^
Varicocele CL	1.34 ± 0.29 ^a,b^		10.2 ± 0.5 ^a^
Varicocele + lycopene	1.29 ± 0.23 ^a,b^	4.7 ± 0.6 ^a,b^	8.3 ± 0.7 ^a,b^
Varicocele + lycopene CL	1.41 ± 0.37 ^a,b^		6.8 ± 0.3 ^a,b^
Varicocele + Se	1.37 ± 0.14 ^a,b^	5.0 ± 0.7 ^a,b^	8.9 ± 0.7 ^a,b^
Varicocele + Se CL	1.47 ± 0.48 ^a,b^		7.4 ± 0.5 ^a,b^
Varicocele + lycopene + Se	1.55 ± 0.32 ^b^	5.6 ± 0.8 ^b^	6.0 ± 0.3 ^b^

**Table 2:** Effects of Se (3 mg/kg/day), lycopene (1 mg/Kg/day), and Se (3 mg/kg/day) plus lycopene (1 mg/Kg/day) on mean tubular diameter (MTD), Johnsen’s score (JS), %TWAC (percentage of tubules with apoptotic cells), and apoptotic index (AI) (mean number of TUNEL-positive cells per tubule) in varicocele rats as compared to sham and varicocele alone rats. Se = selenium; CL = contralateral testis. All values are expressed as mean ± SD; n = 7 animals for each group. ^a^ *p* < 0.05 vs. sham; ^b^ *p* < 0.05 vs. varicocele.

Groups	MTD (µm)	JS	% TWAC
Sham	243 ± 19	9.6 ± 0.4	0.3 ± 0.1
Varicocele	131 ± 25 ^a^	2.9 ± 0.6 ^a^	28.5 ± 6 ^a^
Varicocele CL	173 ± 21 ^a,b^	7.7 ± 0.7 ^a,b^	7.9 ± 3.1 ^a,b^
Varicocele + lycopene	178 ± 15 ^a,b^	7.1 ± 0.9 ^a,b^	11.9 ± 2.4 ^a,b^
Varicocele + lycopene CL	218 ± 19 ^b^	8.9 ± 0.9 ^b^	4.0 ± 1.5 ^a,b^
Varicocele + Se	183 ± 14 ^a,b^	7.4 ± 1.1 ^a,b^	9.3 ± 1.9 ^a,b^
Varicocele + Se CL	224 ± 16 ^b^	9.0 ± 0.4 ^b^	3.8 ± 0.6 ^a,b^
Varicocele + lycopene + Se	238 ± 15 ^b^	9.2 ± 0.7 ^b^	4.1 ± 1.2 ^a,b^
Varicocele + lycopene + Se CL	245 ± 13 ^b^	9.4 ± 0.8 ^b^	1.8 ± 0.4 ^b^

## Data Availability

The data presented in this study are available on request to the corresponding author.
